# Strategies for Improving Case Reports Involving Patients With Rare Diseases

**DOI:** 10.7759/cureus.79864

**Published:** 2025-02-28

**Authors:** Mikaela I Poling, Craig R Dufresne

**Affiliations:** 1 Research, Office of Craig R Dufresne, MD, PC, Fairfax, USA; 2 Plastic and Reconstructive Surgery, Office of Craig R Dufresne, MD, PC, Fairfax, USA

**Keywords:** case report, clinical reasoning, clinical relevance, craniofacial dysostosis, freeman-burian syndrome, freeman-sheldon syndrome, medical writing, rare diseases, research methodology, whistling face syndrome

## Abstract

For those treating patients with rare diseases, there may be a disproportionate clinical reliance on the literature, compared with those treating patients with common problems. Moreover, the rare disease literature consists of a preponderance of case reports. Together, these factors place a higher burden for accuracy on authors of case reports of patients with rare diseases. Our decades of experience with the rare congenital craniofacial myopathy, Freeman-Sheldon syndrome-now, Freeman-Burian syndrome, and other rare diseases suggests that accurate and current information may not efficiently proliferate in the rare disease literature-a potentially significant clinical and scholarly concern. Based on our experience of reading case reports of patients with Freeman-Burian syndrome, we suggest mutually supporting mitigation strategies. Our quality-improvement strategies for rare disease case reports emphasize a careful search of recent literature, not exclusively case reports, in-person clinical experience with the patient described, and involvement of a rare disease expert as bedrocks for improving case report accuracy. We propose that objectively demonstrating the patient’s findings relative to an accepted diagnostic criteria, presenting the clinical course within a known disease mechanism, cautiously proposing a new one, and adhering to the relevant case report guidelines can help construct a stronger case report. We hope the wide dissemination of these quality improvement strategies among authors, editors, peer reviewers, and readers will improve the accuracy and completeness of case reports involving rare diseases to ensure the best chances for advancing clinical care and science for this often marginalized patient population.

## Editorial

Background

Clinicians treating patients with rare diseases, compared with those seeing patients with common complaints, may disproportionately rely on the literature to inform their care for such patients, placing a higher burden for accuracy on authors describing rare diseases. Unfortunately, our decades of experience with one such rare disorder, Freeman-Sheldon syndrome, now Freeman-Burian syndrome (FBS), suggests a concerning apparent lack of proliferation of updated information on rare diseases more generally [1-2]. As we have not encountered others raising the alarm on this issue of potentially significant clinical and scholarly implications for FBS or other rare diseases, we present our experience with FBS and suggest mitigation strategies for potential authors to ensure the best chances for advancing clinical care and science. Editors, peer reviewers, and readers can also use these mitigation strategies to assess the potential value of a manuscript. An early version of this article, from January 26, 2022, is posted to the Authorea preprint server [3].

Of rare disorders, FBS is a good barometer of scholarly informational challenges rare disorders may experience. FBS is quite rare yet relatively well-known and clearly documented clinically and molecularly. FBS is a rare congenital craniofacial myopathy, often involving extra-craniofacial features, especially camptodactyly, equinovarus, and scoliosis; pathognomonic features include: whistling face, microstomia, H or V-shaped cutaneous chin defect, and prominent nasolabial folds. Dominantly transmitted, it is associated with mutations on the head of the embryonic myosin heavy chain gene. Intelligence is unimpaired. Confusion regarding required diagnostic findings and clinically relevant hypotheses dominates the literature, with these types of articles being highly cited [2].

Historically, most articles describing rare diseases are case reports. Since 2020, after the publication of several important articles that began to settle unanswered questions about FBS, we continue to find the accuracy of case reports of patients reported as diagnosed with FBS to have many fundamental flaws involving the definition, classification, inheritance pattern, prevalence, diagnosis, and treatment of this rare disorder. Most errors are shared among multiple articles, with some repeated in all articles. Critiques of each case report are available in our letters to the editor or preprint, for journals not publishing letters to the editor [1]. Our recommendations, based on these findings, may seem axiomatic, but their sincere implementation may provide multiple levels of safeguards to mitigate the risk of presenting inaccurate information in case reports of patients with rare diseases.

Recommendations

Our recommendations for improving the accuracy of case reports involving rare diseases were based on our observations of the literature on FBS and decades of experience with rare disease literature more broadly. These recommendations were written to logically compliment the article development workflow, beginning with background work at initial stages and concluding with quality assurance measures during manuscript drafting (Table 1).

**Table 1:** Recommendations for improving the accuracy of case reports involving rare diseases.

Order of Importance	Recommendations
Patient Care	
1	Only describe patients at least one of the main authors have seen in person.
Literature Search and Background Reading	
2	Before writing a draft, conduct a complete literature search.
a	Review up-to-date information, focusing on the following: narrative reviews, meta-analyses, clinical practice recommendations, studies showing a molecular or genetic cause, reports of accepted diagnostic criteria, and descriptions of treatment.
Rare Disease Expert Review of Case and Presentation	
3	If no authors are an expert on the rare condition, consult an expert.
a	Provide the expert with access to appropriate materials.
Writing the Case Report	
4	In drafting the manuscript, objectively demonstrate how the patient’s findings align with diagnostic criteria.
5	In drafting the manuscript, follow within a known disease mechanism or cautiously propose a new one.
6	In drafting the manuscript, follow CARE Guidelines for Case Reports.

First, author participation in a specific patient care situation should be the inspiration for all case reports, and authors should be familiar with their patient's history, presentation, and care to improve accuracy and precision in reporting. We found that some authors were not familiar with the patient’s history or findings and had a very narrow understanding of their patient’s clinical picture, which led to a poor presentation. 

Second, authors must carry out a thorough literature search and do background reading of up-to-date information, not just focusing on highly-cited articles. We have observed that many of the more highly-cited articles on FBS were outdated or inaccurate. The types of errors we observed in newly published case reports appear to be the scholarly outcome of an insufficient literature search, which would have been prevented by a careful search and review of the literature. In conducting a literature search while considering whether to write a case report of a patient with a rare disease, potential authors should find and carefully read the most recent of as many of the following as possible: a narrative review, meta-analysis, clinical practice recommendations, studies showing a molecular or genetic cause, reports of an accepted diagnostic criteria, and descriptions of treatment. Reviewing mostly case reports, as with outdated articles, led the authors of the articles we reviewed to incorrect conclusions [1]. While high-quality randomized controlled trials and large observational studies were unlikely to be found for many rare diseases, carefully reading a variety of article types should help potential authors.

Third, if no authors were experts on the rare condition, they should seek rare disease experts to review the case and plan the presentation. Authors should provide as much medical data on patients described as possible. These experts also should review any resulting manuscript to evaluate the accuracy of the case presentation, discussion, and appropriateness of the literature cited. Additionally, we considered it ideal but not necessary that the consulting expert see the patient.

Quality assurance steps for manuscript drafting must follow. Fourth, any resulting manuscript should objectively demonstrate how the patient’s findings align with accepted diagnostic criteria. For a rare disease, where neither the majority of authors nor the audience would be expected to be experts, objectively establishing the diagnosis in the context of the accepted diagnostic criteria was paramount. The cogency of the entire case report depended on having established an accurate diagnosis. As with all manuscripts, authors should strive to interpret the relevance of their case and how their case report may point toward future needed research. In the context of rare diseases, this might not be as straightforward and require extra consideration.

Fifth, case reports should follow logically within a known disease mechanism or very cautiously propose a new one. Authors should remember that an unexpected event or feature occurring concomitantly with a rare disease neither implies nor excludes its association with the rare disease. In these cases of unexpected co-occurrences, extra clinical and scholarly investigation and diligence would be required to present a clear picture for the readers to consider for themselves. Finally, while non-specific to rare diseases, authors should carefully adhere to CARE reporting guidelines, which provide minimum standards for published case reports [4].

When choosing to write about a rare disease in the literature, special care must be taken to ensure the most current and accurate information is reviewed and the patient’s presentation is systematically described. While this example was limited to FBS, it was expected similar problems might exist for other rare diseases. An inadequate literature search and repeating inaccurate or outdated information shared by all articles reviewed caused these authors to draw erroneous conclusions, which were passed along to readers [1].

Conclusions

It should be a major goal within medicine to improve the accuracy of case reports describing patients with rare diseases since these case reports often formed the bedrock of literature clinicians depended on to inform their clinical decision-making. For patients with FBS, a lack of improvement in care outcomes was observed, standing as a testament to the outsized deleterious clinical impact poor case reports can have for patients with rare diseases. By following six mutually supporting steps, authors could improve the specific types of accuracy and precision concerns we observed in case reports involving patients with FBS, reducing the kind of low-quality case reports that prompted us to write this editorial and helping to guide the global improvement of care for all patients with rare diseases.
